# Prognostic Significance of Serum Uric Acid and Gamma-Glutamyltransferase in Patients with Advanced Gastric Cancer

**DOI:** 10.1155/2019/1415421

**Published:** 2019-12-06

**Authors:** Shanshan Yang, Xinjia He, Ying Liu, Xiao Ding, Haiping Jiang, Ye Tan, Haijun Lu

**Affiliations:** ^1^Department of Radiation Oncology, Sun Yat-Sen University Cancer Center, State Key Laboratory of Oncology in South China, Collaborative Innovation Center for Cancer Medicine, Guangdong Key Laboratory of Nasopharyngeal Carcinoma Diagnosis and Therapy, Guangzhou, Guangdong, China; ^2^Department of Oncology, The Affiliated Hospital of Qingdao University, Qingdao, Shandong Province, China; ^3^Nursing Department, Jinan Seventh People's Hospital, Jinan, Shandong Province, China

## Abstract

**Purpose:**

In this study, we aim to evaluate the prognostic role of serum uric acid and gamma-glutamyltransferase in advanced gastric cancer patients.

**Methods:**

A total of 180 patients pathologically diagnosed with advanced gastric cancer were included in this retrospective study. We used time-dependent receiver operating characteristic (ROC) curves to identify the optimal cut-off value of serum uric acid (UA) and gamma-glutamyltransferase (GGT). Survival analysis was performed using the Kaplan–Meier method and log-rank test, and multivariate Cox regression analyses were applied. A nomogram was formulated, and the calibration and discrimination of the nomogram were determined by calibration curve and concordance index (C-index). We validated the results using bootstrap resampling and a separate study on 60 patients collected from 2015 to 2017 using the same criteria in other medical center.

**Results:**

Both higher serum uric acid (>228 *μ*mol/L) and higher gamma-glutamyltransferase (>14 U/L) had worse OS and PFS. Univariate analysis indicated that serum uric acid (UA) (*p* < 0.001 and *p* < 0.001) and gamma-glutamyltransferase (GGT) (*p* < 0.001 and *p* = 0.044) were significantly related to overall survival (OS) and progression-free survival (PFS), respectively. Multivariate analysis revealed serum uric acid (UA) and gamma-glutamyltransferase (GGT) were independent prognostic factors for OS (*p* = 0.012, *p* = 0.001). The optimal agreement between actual observation and nomogram prediction was shown by calibration curves. The C-indexes of the nomogram for predicting OS and PFS were 0.748 (95% CI: 0.70-0.79) and 0.728 (95% CI: 0.6741-0.7819), respectively. The results were confirmed in the validation cohort.

**Conclusion:**

We observed that both serum UA and GGT were poor prognostic factors in patients with advanced gastric cancer. And we also formulated and validated a nomogram which can predict individual survival for advanced gastric cancer patients.

## 1. Introduction

Gastric cancer (GC) is one of the most common cancers, rated as the third leading cause of cancer-related death worldwide [1]. Early diagnosis of GC is very difficult, and it is usually diagnosed at an advanced stage, leading to a very low level 5-year survival rate, especially in China [2]. Systemic chemotherapy is an important treatment for advanced gastric cancer (AGC). Despite advancement in oncologic therapies, the prognosis of AGC patients is still very poor, with median overall survival (OS) rarely exceeding 1 year [3].

Recognized independent prognostic factors affecting the survival of AGC include tumor-related factors and systemic inflammatory factors [4]. Various hematological markers, such as neutrophil to lymphocyte ratio (NLR), C-reactive protein (CRP), and plasma fibrinogen levels, have been reported as effective prognostic indicators of AGC [5–8]. However, other laboratory markers which are widely available and inexpensive are also needed.

Uric acid (UA), the product of purine metabolism, derives from oxidation of hypoxanthine and xanthine by the enzyme xanthine oxidoreductase (XOR) in nucleotide metabolism. It is reported that serum uric acid (SUA) is related to various diseases, such as gout, cardiovascular disease, hypertension, acute ischemic stroke, and metabolic syndrome [9–11]. However, the association between uric acid and cancer is not very clear and still remains controversial, and studies on this question are highly limited. While SUA has been regarded to protect against cancer due to its antioxidant feature, some studies reported that hyperuricemia predicts poor survival in several cancers [12].

Gamma-glutamyltransferase (GGT) is an enzyme involved in glutathione (GSH) metabolism [13]. Apart from its function as an indicator of liver diseases, serum GGT is regarded as a sensitive marker in several cancers. It is pointed out that GGT participates in tumor progression through oxidative stress pathways [14]. Both SUA and GGT have been regarded as oxidative stress markers and independent risk factors in cancer incidence.

However, in advanced gastric cancer, the prognostic relevance of SUA as well as GGT has not been elucidated. Accordingly, we aimed to evaluate the prognostic role of SUA and GGT in advanced gastric cancer.

## 2. Materials and Methods

### 2.1. Study Subjects

We performed a retrospective study based on a primary cohort of 180 patients who were pathologically diagnosed with AGC at The Affiliated Hospital of Qingdao University between January 2013 and January 2017. The exclusion criteria consisted of gout, hyperuricemia, cardiovascular disease, diabetes mellitus, metabolic syndrome, hepatic or renal insufficiency, hypertension, infectious disease, other malignancy and patients whose complete clinical data were not available.

From January 2015 to January 2017, an independent cohort of 60 patients pathologically diagnosed with AGC at Qingdao Municipal Hospital formed the validation cohort. And the validation cohort had the same inclusion and exclusion criteria as the primary cohort.

### 2.2. Data Collection

Clinicopathological data were extracted at the time of diagnosis, including age, sex, histopathological records, histologic type, differentiation, T-stage, N-stage, imaging reports, neutrophil, lymphocyte, monocyte, platelet, D-dimer, plasma fibrinogen, serum albumin (ALB), cholesterol, triglyceride, high-density lipoprotein (HDL), carcino-embryonic antigen (CEA), gamma-glutamyltransferase (GGT), uric acid (UA), carbohydrate antigen 19-9 (CA19-9), body mass index (BMI), past history, and family history. The last follow-up date was January 2018. And treatment protocols were obtained during the follow-up period. The study was approved by the Institutional Review Board of The Affiliated Hospital of Qingdao University and Qingdao Municipal Hospital, and all patients provided informed consent.

### 2.3. Statistical Analysis

The time-dependent ROC curves were plotted to identify the optimal cut-off value. Survival curves were drawn using the Kaplan–Meier method and the log-rank test. Univariate and multivariate Cox regression models were used to identify the independent predictors. All statistical analyses were performed using the SPSS 24.0 statistical software program (IBM, USA).

Based on the results of multivariate analysis in primary cohort, a nomogram was plotted by using the package of rms in R version 3.5.1 (https://www.r-project.org/). In the internal validation, the discrimination of the nomogram was assessed by C-index, and the calibration was evaluated by calibration curve which compared nomogram-predicted with actual observed survival probability. The larger the C-index, the more accurate was the prognostic prediction. In the external validation, based on the established nomogram, we calculated the total points of each patient in the validation cohort and performed Cox regression using the total points as a factor; finally, the C-index and calibration curve were obtained according to the regression analysis. *p* < 0.05 was defined as statistically significant.

## 3. Results

### 3.1. Clinicopathologic Characteristics of Patients

In the primary cohort, a total of 180 patients diagnosed with advanced gastric cancer were included. Among them, there were 41 females and 139 males, and the median age was 60 years with a range from 24 to 88 years. The median OS was 11 months (range: 1-49 months). The histologic differentiation was as follows: poorly differentiated (*n* = 160, 88.9%), moderate, or well-differentiated (*n* = 20, 11.1%). Distant lymph node (*n* = 80, 44.4%) was the most common sites of metastases, followed by liver metastasis (*n* = 45, 25%). In the validation cohort, there were 60 patients. The clinicopathologic characteristics of patients in the cohorts are shown in Table 1.

**Figure 1:**
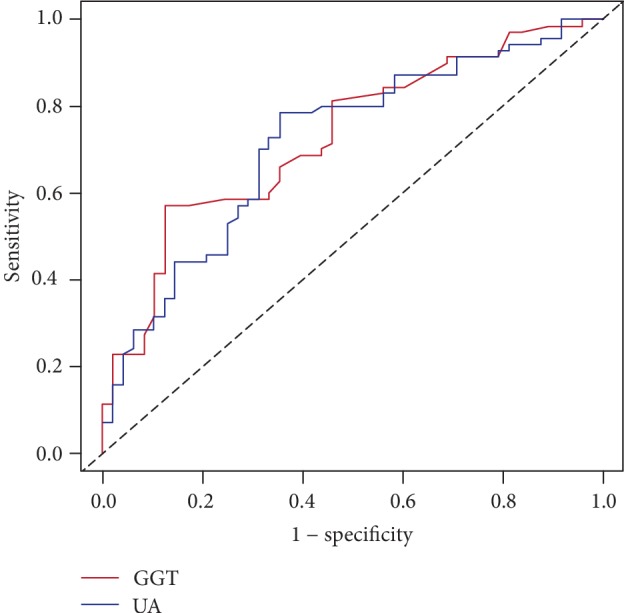
Time-dependent ROC curves for survival prediction. Note: the time-dependent receiver operating characteristic (ROC) curves were plotted to determine the optimal cut-off value.

We performed time-dependent ROC curve to identify the optimal cut-off value based on the largest Youden's index. An SUA of 228 *μ*mol/L calculated by time-dependent ROC curve showed the best specificity and sensitivity, and the area under the curve (AUC) of SUA was 0.72 (95% confidence interval (CI): 0.62-0.81, The cut-off value of GGT was 14 U/L, with an AUC of 0.73 (95% CI: 0.64-0.82; Figure 1).

**Figure 2:**
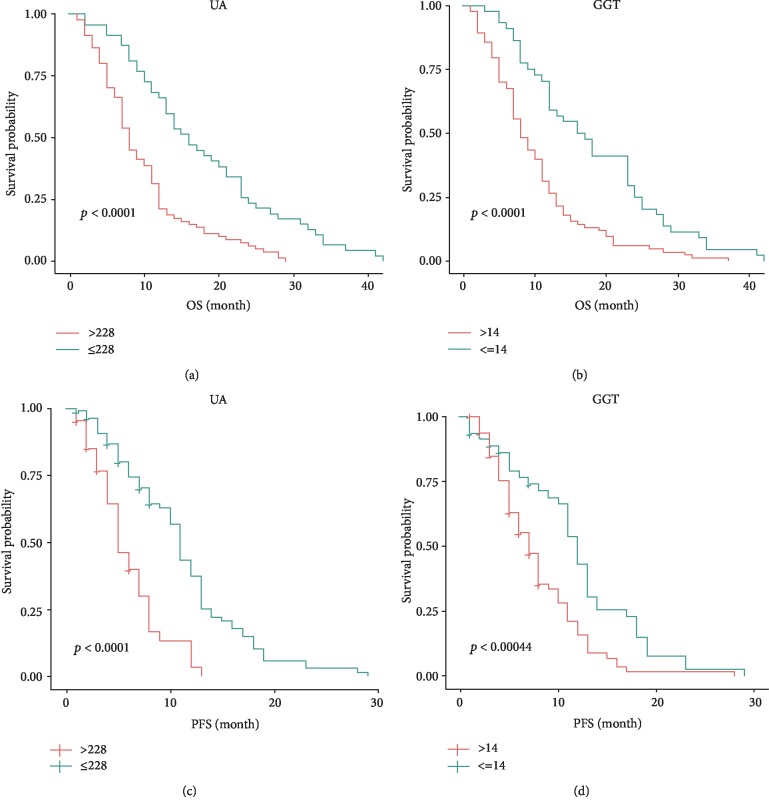
Kaplan–Meier curves for OS and PFS. Notes: (a) OS curves of patients with advanced gastric cancer in the low-SUA group (<228 *μ*mol/L) vs the high-SUA group (≥228 *μ*mol/L), *p*<0.001; (b) OS curves of patients with advanced gastric cancer in the low-GGT group (<14 U/L) vs the high-GGT group (≥14 U/L), *p* < 0.001; (c)PFS curves of patients with advanced gastric cancer in the low-SUA group (<228 *μ*mol/L) vs the high-SUA group (>228 *μ*mol/L), *p* < 0.001; (d) PFS curves of patients with advanced gastric cancer in the low-GGT group (<14 U/L) vs the high-GGT group (>14 U/L), *p* = 0.00044.

As shown in Figure 2, the survival curve of the high SUA group (>228 *μ*mol/L) was different from that of the low group significantly (median OS: 11.00 months vs. 19.00 months, *p* < 0.001, Figure 2(a); median PFS: 7.00 months vs. 11.00 months, *p* < 0.001, Figure 2(c), respectively). And the high GGT patients (>14 U/L) also had a significantly shorter OS and PFS than the low GGT patients (median OS: 11.00 months vs. 23.00 months, *p* < 0.001, Figure 2(b); median PFS: 7.00 months vs. 11.00 months, *p* = 0.0044, Figure 2(d), respectively).

### 3.2. Independent Prognostic Factors in the Primary Cohort

Table 2 shows the results of the univariate analysis in the primary cohort. On multivariate analysis, SUA, CA199, chemotherapy, and TNM stage were significantly independent prognostic factors for OS (*p* = 0.012, *p* = 0.013, *p* < 0.001, and *p* = 0.008) and PFS (*p* < 0.001, *p* < 0.001, *p* = 0.012, and *p* < 0.001). What is more, age, GGT, and lung metastasis were also independent prognostic factors for OS (*p* = 0.004, *p* = 0.001, and *p* = 0.008, respectively; Table 3).

### 3.3. Prognostic Nomogram for OS and PFS

**Figure 3:**
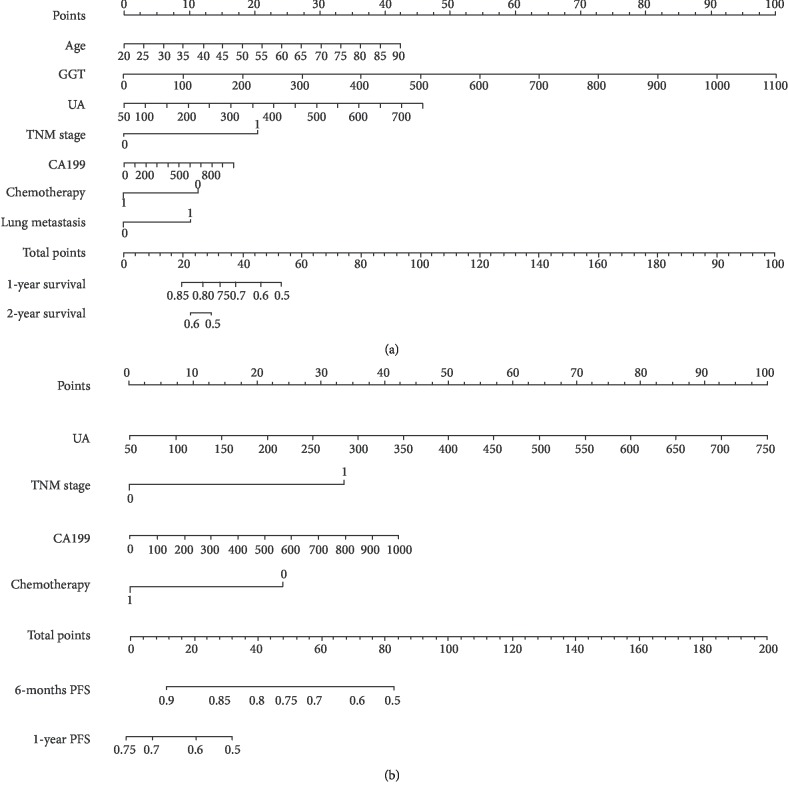
Advanced gastric cancer overall survival (a) and progression-free survival (b) nomogram. Notes: to use the nomogram, an individual patient's value is located on each variable axis, and a line is drawn upward to determine the number of points received for each variable value. The sum of these numbers is located on the total point axis, and a line is drawn downward to the survival axes to determine the likelihood of survival.

**Figure 4:**
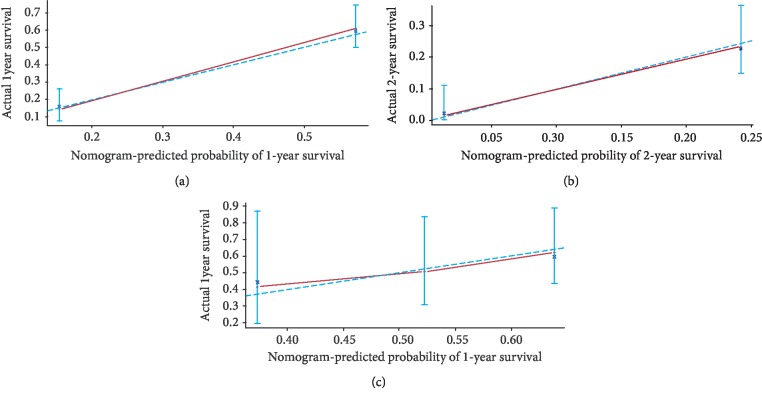
The calibration curve for predicting patient survival at (a) 1 year and (b) 2 years in the primary cohort and at (c) 1 year in the validation cohort. Nomogram-predicted probability of overall survival is plotted on the *x*-axis; actual overall survival is plotted on the *y*-axis.

**Figure 5:**
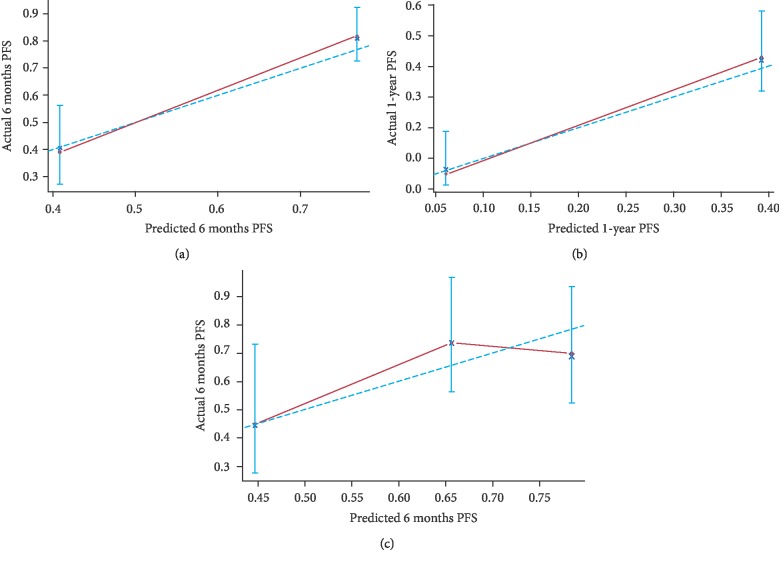
The calibration curve for predicting patient progression-free survival at (a) 6 months and (b) 1 year in the primary cohort and at (c) 6 months in the validation cohort. Nomogram-predicted probability of progression-free survival is plotted on the *x*-axis; actual progression-free survival is plotted on the *y*-axis.

As shown in Figure 3, the prognostic nomogram combined all the important independent factors for OS and PFS in the primary cohort. The Harrell's C-indexes were 0.748 (95% CI: 0.70-0.79) and 0.728 (95% CI: 0.6741-0.7819). The calibration curve for the survival probability indicated an optimal agreement between actual observation and nomogram prediction (Figures 4 and 5).

### 3.4. Calibration and Validation of the Nomogram for OS and PFS

The median OS time was 11.8 months (range: 3-32 months) in the validation cohort. The C-indexes of the nomogram were 0.685 (95% CI: 0.6066-0.7634) and 0.614 (95% CI: 0.50-0.73), and calibration curves for probability of survival revealed optimal agreement between actual observation and nomogram prediction (Figures 4(c) and 5(c)).

## 4. Discussion

In this study, we evaluated the prognostic significance of serum UA and GGT in advanced gastric cancer. We observed that high levels of SUA had worse OS and PFS, the same as GGT. And we also performed a nomogram for predicting survival of AGC patients.

Serum uric acid is a useful marker for diagnosis in many diseases, such as gout, metabolic syndrome, obesity, insulin resistance, T2DM, hypertension, and cardiovascular disease [15–17]. Recently, the correlation between uric acid and cancer has been reported in several studies, which yielded inconsistent findings. Ames et al. firstly made an assumption that uric acid provided an antioxidant defense against cancer. This hypothesis was based on the point that free oxygen radicals were cleared by XOR, thereby protecting against carcinogenesis [12]. Taghizadeh et al. revealed that elevated levels of SUA were related to a low risk of cancer mortality from a large cohort followed up for 38 years [18]. And studies also pointed out the fact that SUA levels were lower in lung cancer and oral cancer patients compared with healthy controls [19, 20]. However, Hiatt and Fireman showed that cancer incidence was not associated with SUA in a large female cohort [21]. Opposite to the protective effect of SUA, some studies demonstrated a rather positive correlation between SUA and cancer, showing that high SUA levels were a risk factor for cancer morbidity and mortality. For example, Petersson et al. proposed that increased uric acid was associated with increased cancer mortality in 1984 [22]. Kolonel et al. found that high levels of uric acid could increase the incidence of prostate cancer [23]. Similarly, in a large prospective study with male and female European cohorts conducted by Strasak et al. [24], it was determined that elevated SUA was significantly related to high risk of cancer mortality (*p* < 0.0001). And Strasak et al. also demonstrated a J-shaped dose-response relationship between SUA and cancer incidence [25]. Furthermore, a meta-analysis elucidated that high levels of SUA increased the risk of cancer incidence and mortality, which differed by gender [26].

Uric acid was also reported as an independent prognostic factor in various cancers. Prior studies have found that elevated levels of SUA predicted a poor survival prognosis in colorectal cancer, non-small-cell lung cancer, pancreatic cancer, esophageal carcinoma, and terminally ill cancer patients [27–31]. On the contrary, in nasopharyngeal carcinoma and colon cancer, high SUA was a favorable prognostic factor [32, 33]. In our study, high pretreatment SUA levels were significantly and independently related to short PFS and OS in advanced gastric cancer.

Several studies also demonstrated that a high level of GGT was associated with higher incidence, recurrence, metastasis and poor prognosis in several cancers. Commonly, serum GGT was regarded as a marker for hepato-biliary tract diseases, especially alcoholic liver disease [34]. Interestingly, several large-scale cohorts studies have indicated that serum GGT was positively related to cancer incidence and site-specific cancers, such as cancers of the respiratory, digestive, and genital organs among both females and males, and GGT was also influenced by environmental and lifestyle factors (such as pollutants, alcohol consumption, smoking, and diet) [35–38]. Simic et al. reported that serum GGT increased in metastatic renal cell carcinoma patients [39]. On the other hand, high GGT was linked to an advanced stage in patients with renal cancer and cervical cancer [40, 41]. In addition, emerging evidence showed that high pretreatment serum GGT levels were correlated to poor prognosis independently in endometrial cancer [42, 43], advanced cervical cancer [44, 45], epithelial ovarian cancer [46], primary metastatic breast cancer [47], gallbladder cancer [48], and nonmetastatic renal cell carcinoma [49].What is more, Wang et al. identified that both serum and tumor GGT levels were poor prognostic factors in patients with gastric cancer, which was in accordance with our results [50].

The mechanism of SUA and GGT in cancer has not been illustrated clearly. It was pointed out that both SUA and GGT were associated with the metabolic syndrome and regarded as oxidative stress markers. And these two markers were independent factors in cancer incidence.

SUA has an antioxidant capacity in the extracellular environment [51] but may also play a dual role as a prooxidant [52]. There are several potential mechanisms. Extracellular UA is proposed to be an antioxidant and a scavenger of hydroxyl radicals and singlet oxygen to protect against cancer [12]. Moreover, dying tumor cells may release uric acid, which can potentiate the immune system against cancer and inhibit tumor cell proliferation and migration [44, 53, 54]. However, contrary to the above hypothesis, high levels of UA have been postulated to have proinflammatory properties that contribute to tumorigenesis. And C-reactive protein, adiponectin, and leptin play an important role in inflammatory environment that are related to SUA and cancer [55]. Furthermore, when UA enters cancer cells, it could inhibit XOR expression which may increase COX-2 levels. Besides, it can also trigger inflammatory stress that is caused by the effects of intracellular UA on COX-2 activation and reactive oxygen species (ROS) generation [55, 56]. Thus, elevated UA levels might promote tumor cell proliferation, migration, and survival. In addition, GGT is also influenced by environmental and lifestyle factors. In our study, increased uric acid was found to be related to poor survival in advanced gastric cancer.

The specific mechanism of GGT in carcinogenesis remains poorly understood. It is uncertain whether GGT has a direct role in tumorigenesis. Serum GGT is related to both inflammation and oxidative stress. Experimental evidence has reported that GGT could regulate crucial redox-sensitive functions, such as cellular proliferative/apoptotic balance. And GGT is a source of ROS during glutathione metabolism, contributing to drug resistance, tumor progression, and invasion [57]. Additionally, it was found that elevated GGT may trigger inflammation in the prostate, because it could modulate the inflammatory mediator [45]. Moreover, elevated GGT may also be correlated with hyperglycemia, which can result in the overproduction of ROS [58]. Finally, GGT may be influenced by environmental and lifestyle factors, which may have direct effects on carcinogenesis [59].

In our study, we evaluated the prognostic value of UA and GGT in AGC patients simultaneously. As we all know, it is important to determine proper cut-off values of UA and GGT. Unlike previous studies, we performed the time-dependent ROC curve to select the proper cut-off values. Also, we have made strict inclusion criteria to exclude the impact of selection bias as possible. With good association with histologic differentiation, GGT and UA had a good association with AGC prognosis. And in multivariate analysis, GGT and UA were independent predictors of OS. What is more, nomograms have been shown to be accurate for predicting cancer prognosis. Thus, a prognostic nomogram for patients with AGC was constructed. The nomogram had a good predictive effect on survival, which was validated by the C-index and the calibration curve.

Our study is believed to be the first to show that both serum UA and GGT are independent factors for the prognosis in advanced gastric cancer. And we constructed a nomogram for predicting survival. Based on this easy-to-use scoring system, physicians could predict an individualized survival. However, there are several limitations in our study. First, a relatively small sample size is a major limitation. Second, this is a retrospective study, and we cannot fully exclude selection bias. What is more, even if we exclude some interference factors, other confounders related to UA and GGT, such as diet, alcohol consumption, and exercise, are not included as variables in this analysis. Besides, all patients included in this study are Chinese. In spite of these limitations, our results indicate that serum UA and GGT could be novel prognostic markers in advanced gastric cancer.

In conclusion, we confirmed that both serum UA and GGT were poor prognostic factors in AGC patients. Besides, we performed a nomogram for predicting an individualized survival of AGC patients. And further prospective and multicenter studies on larger scales are needed to confirm our findings.

## Figures and Tables

**Table 1:** Demographics and clinicopathologic characteristics of patients with advanced gastric cancer.

		Primary cohort		Validation cohort	
		*N* = 180	%	*N* = 60	%
Sex	Female	41	22.8	15	25.0
	Male	139	77.2	45	75.0
Age (years)	Median/range	60.31/(24.00-88.00)		57.52/(24.00-77.00)	
NLR	Median/range	4.61/(0.21-76.29)		5.43/(0.33-76.29)	
PLR	Median/range	207.04/(46.0-1156.4)		211.00/(63.50-632.14)	
LMR	Median/range	4.57/(0.22-84.00)		4.36/(0.43-84.00)	
D-D	Median/range	805.01/(0.22-12690.00)		1367.32/(0.22-12690.00)	
FIB	Median/range	3.85/(0.71-14.00)		4.06/(1.91-14.00)	
ALB	Median/range	36.22/(16.30-59.84)		36.24/(21.30-59.84)	
Cholesterol	Median/range	4.54/(0.82-13.03)		4.39/(0.82-6.87)	
Triglyceride	Median/range	1.10/(0.29-6.83)		1.16/(0.63-1.77)	
HDL	Median/range	1.16/(0.28-2.47)		1.06/(0.36-2.08)	
GGT	Median/range	32.83/(4.00-1076.00)		23.26/(6.00-169.00)	
UA	Median/range	255.94/(56.00-743.53)		269.53/(81.00-634.00)	
CEA	Median/range	66.61/(0.20-1000.00)		50.66/(0.20-1000)	
CA199	Median/range	195.51/(0.60-1000.00)		191.22/(0.60-1000.00)	
BMI	Median/range	22.24/(12.44-30.85)		22.23/(12.44-28.22)	
Histologic differentiation	Moderate/well	20	11.1	7	11.7
	Poor	160	88.9	53	88.3
Tumor location	L	143	79.4	49	81.7
	M	20	11.1	6	10.0
	U	17	9.4	5	8.3
Her-2	Negative	174	96.7	56	93.3
	Positive	6	3.3	4	6.7
Family history	No	175	97.2	58	96.7
	Yes	5	2.8	2	3.3
Surgery	No	122	67.8	35	58.3
	Yes	58	67.8	25	41.7
Chemotherapy	No	40	22.2	4	6.7
	Yes	140	77.8	56	93.3
Radiotherapy	No	167	92.8	56	93.3
	Yes	13	92.8	4	6.7
Target therapy	No	163	90.6	52	86.7
	Yes	17	9.4	8	13.3
Peritoneal metastasis	No	141	78.3	48	80.0
	Yes	39	21.7	12	20.0
Liver metastasis	No	135	75.0	46	76.7
	Yes	45	25.0	14	23.3
Bone metastasis	No	171	95.0	56	93.3
	Yes	9	5.0	4	6.7
Lung metastasis	No	168	93.3	53	88.3
	Yes	12	6.7.	7	11.7.
Ovary metastasis	No	172	95.6	57	95.0
	Yes	8	4.4	3	5.0
Distant lymph node metastasis	No	100	55.6	35	58.3
	Yes	80	44.4	25	41.7
Histologic type	Adenocarcinoma	151	83.9	54	90.0
	Others	29	16.1	6	10.0
T stage	T3	43	23.9	10	16.7
	T4	137	76.1	50	83.3
N stage	N2	33	18.3	15	25.0
	N3	147	81.7	45	75.0
TNM stage	III stage	50	27.8	13	21.7
	IV stage	130	72.2	47	78.3

Abbreviations: AGC: advanced gastric cancer; NLR: neutrophil to lymphocyte ratio; PLR: platelet to lymphocyte ratio; LMR: lymphocyte to monocyte ratio; D-D: D-dimer; FIB: fibrinogen; ALB: albumin; HDL: high-density lipoprotein; GGT: gamma-glutamyltransferase; UA: uric acid; CEA: carcino-embryonic antigen; BMI: body mass index.

**Table 2:** Univariate analysis of primary cohort (*N* = 180).

			OS			PFS	
		*p*	HR	95% CI	*p*	HR	95% CI
Sex	(Male vs Female)	0.599	1.113	0.746-1.663	0.117	1.371	0.924-2.033
Age		0.001	1.030	1.013-1.048	0.403	1.007	0.991-1.022
NLR		0.021	1.025	1.004-1.047	0.036	1.029	1.002-1.057
PLR		0.014	1.001	1.000-1.002	0.170	1.001	1.000-1.002
LMR		0.965	1.000	0.979-1.023	0.610	0.994	0.971-1.018
D-D		0.153	1.000	1.000-1.000	0.019	1.000	1.000-1.000
FIB		0.265	1.053	0.962-1.152	0.019	1.098	1.016-1.187
ALB		0.021	0.962	0.931-0.994	0.102	0.975	0.945-1.005
Cholesterol		0.346	0.933	0.807-1.078	0.560	0.963	0.847-1.094
Triglyceride		0.420	1.089	0.885-1.341	0.994	1.001	0.821-1.220
HDL		<0.001	0.328	0.181-0.594	0.019	0.542	0.324-0.906
GGT		<0.001	1.006	1.003-1.009	0.044	1.003	1.000-1.006
UA		<0.001	1.003	1.002-1.005	<0.001	1.004	1.002-1.006
CEA		0.004	1.001	1.000-1.002	0.122	1.001	1.000-1.002
CA199		0.002	1.001	1.000-1.001	<0.001	1.001	1.000-1.001
BMI		0.472	0.981	0.932-1.033	0.733	1.008	0.961-1.057
Histologic differentiation (poor vs moderate/well)		0.442	1.223	0.732-2.042	0.488	1.187	0.732-1.925
Tumor location	L						
	M	0.099	0.597	0.324-1.103	0.28	0.749	0.443-1.266
	U	0.552	0.843	0.481-1.479	0.872	0.958	0.567-1.618
Her2	(Yes vs no)	0.942	0.958	0.303-3.024	0.346	1.485	0.653-3.38
Family history	(Yes vs no)	0.272	0.524	0.165-1.662	0.514	0.742	0.303-1.818
Surgery	(Yes vs no)	0.009	0.6	0.41-0.878	0.041	0.696	0.492-0.985
Chemotherapy	(Yes vs no)	<0.001	0.355	0.242-0.521	0.04	0.643	0.422-0.98
Radiotherapy	(Yes vs no)	0.196	0.663	0.356-1.236	0.107	0.624	0.352-1.107
Target therapy	(Yes vs no)	0.023	0.434	0.212-0.892	0.49	0.828	0.484-1.416
Peritoneal metastasis	(Yes vs no)	0.291	1.251	0.825-1.896	0.496	1.141	0.78-1.669
Liver metastasis	(Yes vs no)	0.839	0.961	0.652-1.415	0.297	0.82	0.565-1.19
Bone metastasis	(Yes vs no)	0.751	0.875	0.385-1.991	0.703	0.862	0.403-1.845
Lung metastasis	(Yes vs no)	0.017	2.076	1.137-3.789	0.436	1.292	0.678-2.465
Ovary metastasis (yes vs no)		0.75	1.133	0.524-2.451	0.65	0.827	0.364-1.879
Distant lymph node metastasis	(Yes vs no)	0.031	1.475	1.037-2.1	0.366	1.162	0.839-1.608
Histologic type (others v adenocarcinoma)		0.372	1.240	0.773-1.990	0.240	1.311	0.835-2.060
T stage	(T4 vs T3)	0.711	0.919	0.590-1.434	0.011	0.616	0.424-0.895
N stage	(N3 vs N2)	0.741	1.078	0.691-1.680	0.706	1.083	0.717-1.635
TNM stage	(IV vs III)	0.004	1.750	1.194-2.565	<0.001	2.222	1.529-3.227

Abbreviations: NLR: neutrophil to lymphocyte ratio; PLR: platelet to lymphocyte ratio; LMR: lymphocyte to monocyte ratio; D-D: D-dimer; FIB: fibrinogen; ALB: albumin; HDL: high-density lipoprotein; GGT: gamma-glutamyltransferase; UA: uric acid; CEA: carcino-embryonic antigen; BMI: body mass index.

**Table 3:** Multivariate analysis of primary cohort (*N* = 180).

			OS			PFS	
		*p*	HR	95% CI	*p*	HR	95% CI
Age		0.004	1.026	1.008-1.044			
NLR		0.138	1.022	0.993-1.052	0.079	1.027	0.997-1.057
DD					0.205	1.000	1.000-1.000
FIB					0.537	1.031	0.937-1.134
PLR		0.775	1.000	0.999-1.001	0.365	0.762	0.423-1.372
ALB		0.732	0.994	0.962-1.027			
HDL		0.273	0.707	0.380-1.314	0.365	0.762	0.423-1.372
GGT		0.001	1.004	1.002-1.007	0.858	1.000	0.995-1.004
UA		0.012	1.002	1.000-1.004	<0.001	1.003	1.002-1.005
CEA		0.835	1.000	0.999-1.001			
CA199		0.013	1.001	1.000-1.001	<0.001	1.001	1.000-1.001
Surgery	(Yes vs no)	0.509	0.862	0.555-1.339	0.456	0.859	0.576-1.281
Chemotherapy	(Yes vs no)	<0.001	0.416	0.277-0.626	0.012	0.576	0.374-0.887
Target therapy	(Yes vs no)	0.239	0.623	0.284-1.369			
Lung metastasis	(Yes vs no)	0.008	2.290	1.242-4.222			
Distant lymph node metastasis	(Yes vs no)	0.087	1.402	0.953-2.063			
T stage					0.257	0.790	0.524-1.188
TNM stage	(IV vs III)	0.008	1.720	1.150-2.571	<0.001	2.058	1.408-3.009

Abbreviations: NLR: neutrophil to lymphocyte ratio; PLR: platelet to lymphocyte ratio; LMR: lymphocyte to monocyte ratio; D-D: D-dimer; FIB: fibrinogen; ALB: albumin; HDL: high-density lipoprotein; GGT: gamma-glutamyltransferase; UA: uric acid; CEA: carcino-embryonic antigen; BMI: body mass index.

## Data Availability

The retrospective data used to support the findings of this study are included within the article partially, and the whole data will be available from the corresponding author upon request after publication.

## Abbreviations

NLR: Neutrophil to lymphocyte ratio
PLR: Platelet to lymphocyte ratio
LMR: Lymphocyte to monocyte ratio
D-D: D-dimer
FIB: Fibrinogen
ALB: Albumin
HDL: High-density lipoprotein
GGT: Gamma-glutamyltransferase
UA: Uric acid
CEA: Carcino-embryonic antigen
BMI: Body mass index
